# Subalgebras of BCK/BCI-Algebras Based on Cubic Soft Sets

**DOI:** 10.1155/2014/458638

**Published:** 2014-05-08

**Authors:** G. Muhiuddin, Feng Feng, Young Bae Jun

**Affiliations:** ^1^Department of Mathematics, University of Tabuk, Tabuk 71491, Saudi Arabia; ^2^Department of Applied Mathematics, School of Science, Xian University of Posts and Telecommunications, Xian 710121, China; ^3^Department of Mathematics Education, Gyeongsang National University, Jinju 660-701, Republic of Korea

## Abstract

Operations of cubic soft sets including “AND” operation and “OR” operation based on *P*-orders and *R*-orders are introduced and some related properties are investigated. An example is presented to show that the *R*-union of two internal cubic soft sets might not be internal. A sufficient condition is provided, which ensure that the *R*-union of two internal cubic soft sets is also internal. Moreover, some properties of cubic soft subalgebras of BCK/BCI-algebras based on a given parameter are discussed.

## 1. Introduction

Zadeh [1] made an extension of the concept of a fuzzy set by an interval-valued fuzzy set, that is, a fuzzy set with an interval-valued membership function. Using a fuzzy set and an interval-valued fuzzy set, Jun et al. [2] introduced a new notion, called a (internal, external) cubic set, and investigated several properties. They dealt with *P*-union, *P*-intersection, *R*-union, and *R*-intersection of cubic sets and investigated several related properties. Later on, Jun et al. [3] applied the notion of cubic set theory to BCI-algebras.

To solve complicated problems in economics, engineering, and environment, we cannot successfully make use of classical methods because of various uncertainties typical for those real-word problems. On the contrary, uncertainties could be dealt with the help of a wide range of contemporary mathematical theories such as probability theory, theory of fuzzy sets [4], interval mathematics [5], and rough set theory [6, 7]. However, all of these theories have their own difficulties which were pointed out in [8]. Further, Maji et al. [9] and Molodtsov [8] suggested that one reason for these difficulties might be due to the inadequacy of the parameterization tool of the theory. To overcome these difficulties, Molodtsov [8] introduced the concept of soft set as a new mathematical tool for dealing with uncertainties based on the viewpoint of parameterization. It has been demonstrated that soft sets have potential applications in various fields such as the smoothness of functions, game theory, operations research, Riemann integration, Perron integration, probability theory, and measurement theory [8, 10]. Since then, many researchers around the world have contributed to soft set theory from various aspects [9, 11–15]. Soft set based decision making was first considered by Maji et al. [9]. Çağman and Enginoglu [16] developed the *uni*-*int* decision making method in virtue of soft sets. Feng et al. [17] improve and further extend Çağman and Enginoglu's approach using choice value soft sets and k-satisfaction relations. It is interesting to see that soft sets are closely related to many other soft computing models such as rough sets and fuzzy sets [18, 19]. Aktaş and Çağman [20] defined the notion of soft groups and derived some related properties. This initiated an important research direction concerning algebraic properties of soft sets in miscellaneous kinds of algebras such as BCK/BCI-algberas [21], *d*-algebras [22], semirings [23], rings [24], Lie algebras [25], and *K*-algebras [26, 27]. In addition, Feng and Li [28] ascertained the relationships among five different types of soft subsets and considered the free soft algebras associated with soft product operations. It has been shown that soft sets have some nonclassical algebraic properties which are distinct from those of crisp sets or fuzzy sets.

Recently, combining cubic sets and soft sets, the first author together with Al-roqi [29] introduced the notions of (internal, external) cubic soft sets, *P*-cubic (resp., *R*-cubic) soft subsets, *R*-union (resp., *R*-intersection, *P*-union, and *P*-intersection) of cubic soft sets, and the complement of a cubic soft set. They investigated several related properties and applied the notion of cubic soft sets to BCK/BCI-algebras.

In this paper, we consider several basic operations of cubic soft sets, namely, “AND” operation and “OR” operation based on the *P*-order and the *R*-order. We provide an example to illustrate that the *R*-union of two internal cubic soft sets might not be internal. Then we discuss the condition for the *R*-union of two internal cubic soft sets to be an internal cubic soft set. We also investigate several properties of cubic soft subalgebras of BCK/BCI-algebras based on a given parameter.

## 2. Preliminary

In this section we include some elementary aspects that are necessary for this paper.

An algebra (*X*; ∗, 0) of type (2,0) is called a BCI-algebra if it satisfies the following axioms:

- (∀*x*, *y*, *z* ∈ *X*)  (((*x*∗*y*)∗(*x*∗*z*))∗(*z*∗*y*) = 0),
- (∀*x*, *y* ∈ *X*)  ((*x*∗(*x*∗*y*))∗*y* = 0),
- (∀*x* ∈ *X*)  (*x*∗*x* = 0),
- (∀*x*, *y* ∈ *X*)  (*x*∗*y* = 0, *y*∗*x* = 0⇒*x* = *y*).

If a BCI-algebra *X* satisfies the following identity:

(v) (∀*x* ∈ *X*)  (0∗*x* = 0),

then *X* is called a BCK*-*algebra. Any BCK/BCI-algebra *X* satisfies the following conditions:

(a1) (∀*x* ∈ *X*)  (*x*∗0 = *x*),
(a2) (∀*x*, *y*, *z* ∈ *X*)  (*x*∗*y* = 0⇒(*x*∗*z*)∗(*y*∗*z*) = 0, (*z*∗*y*)∗(*z*∗*x*) = 0),
(a3) (∀*x*, *y*, *z* ∈ *X*)  ((*x*∗*y*)∗*z* = (*x*∗*z*)∗*y*),
(a4) (∀*x*, *y*, *z* ∈ *X*)  (((*x*∗*z*)∗(*y*∗*z*))∗(*x*∗*y*) = 0).

A* fuzzy set* in a set *X* is defined to be a function *λ* : *X* → *I*, where *I* = [0,1]. Denote by *I* ^*X*^ the collection of all fuzzy sets in a set *X*. Define a relation ≤ on *I* ^*X*^ as follows:

(1) $$\begin{array}{c} \left(\forall \lambda ,\mu \in I^{X}\right)\;\left(\lambda \leq \mu ⟺\left(\forall x\in X\right)\left(\lambda \left(x\right)\leq \mu \left(x\right)\right)\right). \end{array}$$

The join (∨) and meet (∧) of *λ* and *μ* are defined by

(2) $$\begin{array}{c} \left(\lambda \vee \mu \right)\left(x\right)=\max\left\{\lambda \left(x\right),\mu \left(x\right)\right\},\\ \left(\lambda \wedge \mu \right)\left(x\right)=\min\left\{\lambda \left(x\right),\mu \left(x\right)\right\}, \end{array}$$

respectively, for all *x* ∈ *X*. The complement of *λ*, denoted by *λ* ^*c*^, is defined by

(3) $$\begin{array}{c} \left(\forall x\in X\right)\;\left(\lambda^{c}\left(x\right)=1-\lambda \left(x\right)\right). \end{array}$$

For a family {*λ*_*i*_ | *i* ∈ Λ} of fuzzy sets in *X*, we define the join (∨) and meet (∧) operations as follows:

(4) $$\begin{array}{c} \left(\overset{}{\underset{i\in \Lambda }{⋁}}\lambda_{i}\right)\left(x\right)=\sup\left\{\lambda_{i}\left(x\right)\;|\;i\in \Lambda \right\},\\ \left(\overset{}{\underset{i\in \Lambda }{⋀}}\lambda_{i}\right)\left(x\right)=\inf\left\{\lambda_{i}\left(x\right)\;|\;i\in \Lambda \right\}, \end{array}$$

respectively, for all *x* ∈ *X*.

By an* interval number* we mean a closed subinterval $\tilde{a}=\left[a^{-},a^{+}\right]$ of *I*, where 0 ≤ *a* ^−^ ≤ *a* ^+^ ≤ 1. Denote by [*I*] the set of all interval numbers. Let us define what is known as* refined minimum* and* refined maximum* (briefly, *r*min⁡ and *r*max⁡) of two elements in [*I*]. We also define the symbols “⪰,” “⪯,” and “ = ” in case of two elements in [*I*]. Consider two interval numbers ${\tilde{a}}_{1}:=\left[a_{1}^{-},a_{1}^{+}\right]$ and ${\tilde{a}}_{2}:=\left[a_{2}^{-},a_{2}^{+}\right]$. Then,

(5) $$\begin{array}{c} r\min\left\{{\tilde{a}}_{1},{\tilde{a}}_{2}\right\}=\left[\min\left\{a_{1}^{-},a_{2}^{-}\right\},\min\left\{a_{1}^{+},a_{2}^{+}\right\}\right],\\ r\max\left\{{\tilde{a}}_{1},{\tilde{a}}_{2}\right\}=\left[\max\left\{a_{1}^{-},a_{2}^{-}\right\},\max\left\{a_{1}^{+},a_{2}^{+}\right\}\right],\\ {\tilde{a}}_{1}⪰{\tilde{a}}_{2}\;\text{iff}\;a_{1}^{-}\geq a_{2}^{-},a_{1}^{+}\geq a_{2}^{+}, \end{array}$$

and similarly we may have ${\tilde{a}}_{1}⪯{\tilde{a}}_{2}$ and ${\tilde{a}}_{1}={\tilde{a}}_{2}$. To say ${\tilde{a}}_{1}≻{\tilde{a}}_{2}$ (resp., ${\tilde{a}}_{1}≺{\tilde{a}}_{2}$), we mean ${\tilde{a}}_{1}⪰{\tilde{a}}_{2}$ and ${\tilde{a}}_{1}\neq {\tilde{a}}_{2}$ (resp., ${\tilde{a}}_{1}⪯{\tilde{a}}_{2}$ and ${\tilde{a}}_{1}\neq {\tilde{a}}_{2}$). Let ${\tilde{a}}_{i}\in [I]$, where *i* ∈ Λ. We define

(6) $$\begin{array}{c} \underset{i\in \Lambda }{r\inf\;}{\tilde{a}}_{i}=\left[\underset{i\in \Lambda }{\inf}\;a_{i}^{-},\underset{i\in \Lambda }{\inf}\;a_{i}^{+}\right],\\ \underset{i\in \Lambda }{r\sup\;}{\tilde{a}}_{i}=\left[\underset{i\in \Lambda }{\sup}\;a_{i}^{-},\underset{i\in \Lambda }{\sup}\;a_{i}^{+}\right]. \end{array}$$

For any $\tilde{a}\in [I]$, its* complement*, denoted by ${\tilde{a}}^{c}$, is defined to be the interval number:

(7) $$\begin{array}{c} {\tilde{a}}^{c}=\left[1-a^{+},1-a^{-}\right]. \end{array}$$

Let *X* be a nonempty set. A function *A* : *X* → [*I*] is called an* interval-valued fuzzy set* (briefly, an IVF* set*) in *X*. Let [*I*]^*X*^ stand for the set of all IVF sets in *X*. For every *A* ∈ [*I*]^*X*^ and *x* ∈ *X*, *A*(*x*) = [*A* ^−^(*x*), *A* ^+^(*x*)] is called the* degree* of membership of an element *x* to *A*, where *A* ^−^ : *X* → *I* and *A* ^+^ : *X* → *I* are fuzzy sets in *X* which are called a* lower fuzzy set* and an* upper fuzzy set* in *X*, respectively. For simplicity, we denote *A* = [*A* ^−^, *A* ^+^]. For every *A*, *B* ∈ [*I*]^*X*^, we define

(8) $$\begin{array}{c} A\subseteq B⟺A\left(x\right)⪯B\left(x\right)\;\forall x\in X,\\ A=B⟺A\left(x\right)=B\left(x\right)\;\forall x\in X. \end{array}$$

The complement *A* ^*c*^ of *A* ∈ [*I*]^*X*^ is defined as follows: *A* ^*c*^(*x*) = *A*(*x*)^*c*^ for all *x* ∈ *X*; that is,

(9) $$\begin{array}{c} A^{c}\left(x\right)=\left[1-A^{+}\left(x\right),1-A^{-}\left(x\right)\right]\;\forall x\in X. \end{array}$$

For a family {*A*_*i*_ | *i* ∈ Λ} of IVF sets in *X* where Λ is an index set, the* union G* = ⋃_*i*∈Λ_ *A*_*i*_ and the* intersection F* = ⋂_*i*∈Λ_ *A*_*i*_ are defined as follows:

(10) $$\begin{array}{c} G\left(x\right)=\left(\overset{}{\underset{i\in \Lambda }{⋃}}A_{i}\right)\left(x\right)=r\;\underset{i\in \Lambda }{\sup}\;A_{i}\left(x\right),\\ F\left(x\right)=\left(\overset{}{\underset{i\in \Lambda }{⋂}}A_{i}\right)\left(x\right)=r\;\underset{i\in \Lambda }{\inf}\;A_{i}\left(x\right) \end{array}$$

for all *x* ∈ *X*, respectively.

Molodtsov [8] defined the soft set in the following way: let *U* be an initial universe set and let *E* be a set of parameters. Let *P*(*U*) denote the power set of *U* and *A* ⊂ *E*.

A pair (*F*, *A*) is called a* soft set* over *U*, where *F* is a mapping given by

(11) $$\begin{array}{c} F:A⟶P\left(U\right). \end{array}$$

In other words, a soft set over *U* is a parameterized family of subsets of the universe *U*. For *ɛ* ∈ *A*, *F*(*ɛ*) may be considered as the set of *ɛ*-approximate elements of the soft set (*F*, *A*). Clearly, a soft set is not a set. For illustration, Molodtsov considered several examples in [8].

## 3. Cubic Soft Sets

**Definition (see [2])**

Let *U* be a universe. By a* cubic set* in *U* one means a structure

(12) $$\begin{array}{c} A=\left\{\langle x,A\left(x\right),\lambda \left(x\right)\rangle \;|\;x\in U\right\} \end{array}$$

in which *A* is an IVF set in *U* and *λ* is a fuzzy set in *U*.

**Definition (see [2])**

Let *A* = 〈*A*, *λ*〉 and *B* = 〈*B*, *μ*〉 be cubic sets in a universe *U*. Then one defines the following.

- (Equality) *A* = *B*⇔*A* = *B* and *λ* = *μ*.
- (*P*-order) *A* ⊆_*P*_ *B*⇔*A*⊆*B* and *λ* ≤ *μ*.
- (*R*-order) *A* ⊆_*R*_ *B*⇔*A*⊆*B* and *λ* ≥ *μ*.

**Definition 3 (see [2])**

For any *A*_*i*_ = {〈*x*, *A*_*i*_(*x*), *λ*_*i*_(*x*)〉∣*x* ∈ *U*} where *i* ∈ Λ, one defines

-  ⋃_*P*,  *i*∈Λ_ *A*_*i*_ = {〈*x*, (⋃_*i*∈Λ_ *A*_*i*_)(*x*), (∨_*i*∈Λ_ *λ*_*i*_)(*x*)〉 | *x* ∈ *U*} (*P*-union);
- ⋂_*P*,  *i*∈Λ_ *A*_*i*_ = {〈*x*, (⋂_*i*∈Λ_ *A*_*i*_)(*x*), (∧_*i*∈Λ_ *λ*_*i*_)(*x*)〉 | *x* ∈ *U*} (*P*-intersection);
- ⋃_*R*,  *i*∈Λ_ *A*_*i*_ = {〈*x*, (⋃_*i*∈Λ_ *A*_*i*_)(*x*), (∧_*i*∈Λ_ *λ*_*i*_)(*x*)〉 | *x* ∈ *U*} (*R*-union);
- ⋂_*R*,  *i*∈Λ_ *A*_*i*_ = {〈*x*, (⋂_*i*∈Λ_ *A*_*i*_)(*x*), (∨_*i*∈Λ_ *λ*_*i*_)(*x*)〉 | *x* ∈ *U*} (*R*-intersection).

The complement of *A* = 〈*A*, *λ*〉 is defined to be the cubic soft set:

(13) $$\begin{array}{c} A^{c}=\left\{\langle x,A^{c}\left(x\right),1-\lambda \left(x\right)\rangle \;|\;x\in U\right\}. \end{array}$$

Obviously, ${\left(A^{c}\right)}^{c}=A,\;{\hat{0}}^{c}=\hat{1}$, ${\hat{1}}^{c}=\hat{0}$, ${\ddot{0}}^{c}=\ddot{1}$, and ${\ddot{1}}^{c}=\ddot{0}$. For any

(14) $$\begin{array}{c} A_{i}=\left\{\langle x,A_{i}\left(x\right),\lambda_{i}\left(x\right)\rangle \;|\;x\in U\right\},\;i\in \Lambda , \end{array}$$

we have (⋃_*P*,  *i*∈Λ_ *A*_*i*_)^*c*^ = ⋃_*P*,  *i*∈Λ_(*A*_*i*_)^*c*^ and (⋃_*P*,  *i*∈Λ_ *A*_*i*_)^*c*^ = ⋃_*P*,  *i*∈Λ_(*A*_*i*_)^*c*^. Also we have

(15) (⋃i∈ΛAiR)c=⋂ i∈Λ(Ai)cR,(⋂i∈ΛAiR)c=⋃ i∈Λ(Ai)cR.

In what follows, a cubic set $A=\left\{\langle x,{\bar{\mu }}_{A}(x),\lambda_{A}(x)\rangle |x\in U\right\}$ is simply denoted by $A=\langle {\bar{\mu }}_{A},\lambda_{A}\rangle$, and denote by *C* ^*U*^ the collection of all cubic sets in *U*.

**Definition 4 (see [29])**

Let *U* be an initial universe set and let *E* be a set of parameters. A* cubic soft set* over *U* is defined to be a pair (*F*, *A*) where *F* is a mapping from *A* to *C* ^*U*^ and *A* ⊂ *E*. Note that the pair (*F*, *A*) can be represented as the following set:

(16) $$\begin{array}{c} \left(F,A\right)∶=\left\{F\left(e\right)\;|\;e\in A\right\},\;\text{where}\;\;F\left(e\right)=\langle {\bar{\mu }}_{F(e)},\lambda_{F(e)}\rangle .\; \end{array}$$

**Definition 5**

Let (*F*, *A*) and (*G*, *B*) be cubic soft sets over *U*. Then “(*F*, *A*) AND (*G*, *B*) based on the *P*-order” is denoted by $\left(F,A\right){\tilde{\wedge }}_{P}\;(G,B)$ and is defined by

(17) $$\begin{array}{c} \left(F,A\right)\underset{P}{\tilde{⋀}}\left(G,B\right)=\left(H,A\times B\right), \end{array}$$

where *H*(*ɛ*_*A*_, *ɛ*_*B*_) = *F*(*ɛ*_*A*_) ⋂_*P*_ *G*(*ɛ*_*B*_)  for all (*ɛ*_*A*_, *ɛ*_*B*_) ∈ *A* × *B*.

**Definition 6**

Let (*F*, *A*) and (*G*, *B*) be cubic soft sets over *U*. Then “(*F*, *A*) AND (*G*, *B*) based on the *R*-order” is denoted by $\left(F,A){\tilde{\wedge }}_{R}(G,B\right)$ and is defined by

(18) $$\begin{array}{c} \left(F,A\right)\underset{R}{\tilde{⋀}}\left(G,B\right)=\left(H,A\times B\right), \end{array}$$

where *H*(*ɛ*_*A*_, *ɛ*_*B*_) = *F*(*ɛ*_*A*_)⋂_*R*_ *G*(*ɛ*_*B*_) for all (*ɛ*_*A*_, *ɛ*_*B*_) ∈ *A* × *B*.

**Definition 7**

Let (*F*, *A*) and (*G*, *B*) be cubic soft sets over *U*. Then “(*F*, *A*) OR (*G*, *B*) based on the *P*-order” is denoted by $\left(F,A){\tilde{\vee }}_{P}(G,B\right)$ and is defined by

(19) $$\begin{array}{c} \left(F,A\right)\underset{P}{\tilde{⋁}}\left(G,B\right)=\left(H,A\times B\right), \end{array}$$

where *H*(*ɛ*_*A*_, *ɛ*_*B*_) = *F*(*ɛ*_*A*_)⋃_*P*_ *G*(*ɛ*_*B*_)  for all (*ɛ*_*A*_, *ɛ*_*B*_) ∈ *A* × *B*.

**Definition 8**

Let (*F*, *A*) and (*G*, *B*) be cubic soft sets over *U*. Then “(*F*, *A*) OR (*G*, *B*) based on the *R*-order” is denoted by $\left(F,A){\tilde{\vee }}_{R}(G,B\right)$ and is defined by

(20) $$\begin{array}{c} \left(F,A\right)\underset{R}{\tilde{⋁}}\left(G,B\right)=\left(H,A\times B\right), \end{array}$$

where *H*(*ɛ*_*A*_, *ɛ*_*B*_) = *F*(*ɛ*_*A*_) ⋃_*R*_ *G*(*ɛ*_*B*_) for all (*ɛ*_*A*_, *ɛ*_*B*_) ∈ *A* × *B*.

**Example 9**

Suppose that there are six houses in the universe *U* given by *U* = {*h*_1_, *h*_2_, *h*_3_, *h*_4_, *h*_5_, *h*_6_} and *E* = {*e*_1_, *e*_2_, *e*_3_, *e*_4_, *e*_5_}, where

 
*e*_1_ stands for the parameter “expensive,”
 
*e*_2_ stands for the parameter “beautiful,”
 
*e*_3_ stands for the parameter “wooden,”
 
*e*_4_ stands for the parameter “cheap,”
 
*e*_5_ stands for the parameter “in the green surroundings.”

For *A* = {*e*_1_, *e*_3_, *e*_4_}⊆*E*, the set (*F*, *A*)∶ = {*F*(*e*_1_), *F*(*e*_3_), *F*(*e*_4_)} is a cubic soft set over *U* where

(21) F(e1)={〈h1,[0.5,0.8],0.6〉,〈h2,[1,1],0.7〉,〈h3,[0.1,0.7],0.5〉,〈h4,[0.2,0.6],0.9〉,〈h5,[0.3,0.9],0.4〉,〈h6,[0.2,0.3],0.3〉},F(e3)={〈h1,[0.2,0.5],0.3〉,〈h2,[0.3,0.6],0.7〉,〈h3,[0.1,0.2],0.4〉,〈h4,[0.2,0.7],0.2〉,〈h5,[0.7,0.9],0.5〉,〈h6,[0.3,0.5],0.3〉},F(e4)={〈h1,[0.4,0.6],0.7〉,〈h2,[0.1,0.2],0.7〉,〈h3,[0.1,0.7],0.3〉,〈h4,[0.3,0.6],0.2〉,〈h5,[0.4,0.8],0.7〉,〈h6,[0.6,0.7],0.8〉}.

The cubic soft set (*F*, *A*) can be represented in the tabular form of Table 1 (see [29]).

For a subset *B* = {*e*_2_, *e*_5_}⊆*E*, consider the cubic soft set (*G*, *B*) with the tabular representation in Table 2.

(1) “(*F*, *A*) OR (*G*, *B*) based on the *P*-order” is a soft set
  (22) $$\begin{array}{c} \left(F,A\right)\underset{P}{\tilde{⋁}}\left(G,B\right)=\left(H,A\times B\right) \end{array}$$
  with the tabular representation in Table 3.
(2) “(*F*, *A*) OR (*G*, *B*) based on the *R*-order” is a soft set
  (23) $$\begin{array}{c} \left(F,A\right)\underset{R}{\tilde{⋁}}\left(G,B\right)=\left(H,A\times B\right) \end{array}$$
  with the tabular representation in Table 4.
(3) “(*F*, *A*) AND (*G*, *B*) based on the *P*-order” is a soft set
  (24) $$\begin{array}{c} \left(F,A\right)\underset{P}{\tilde{⋀}}\left(G,B\right)=\left(H,A\times B\right) \end{array}$$
  with the tabular representation in Table 5.
(4) “(*F*, *A*) AND (*G*, *B*) based on the *R*-order” is a soft set
  (25) $$\begin{array}{c} \left(F,A\right)\underset{R}{\tilde{⋀}}\left(G,B\right)=\left(H,A\times B\right) \end{array}$$
  with the tabular representation in Table 6.

In [29], Muhiuddin and Al-roqi posed the following question.

**Question 1**

Is the *R*-union of two internal cubic soft sets an internal cubic soft set?

The following example shows that the answer to this question is negative.

**Example 10**

Suppose that there are three houses in the universe *U* given by *U* = {*h*_1_, *h*_2_, *h*_3_} and *E* = {*e*_1_, *e*_2_, *e*_3_, *e*_4_, *e*_5_} is the set of parameters, where

 
*e*_1_ stands for the parameter “expensive,”
 
*e*_2_ stands for the parameter “beautiful,”
 
*e*_3_ stands for the parameter “wooden,”
 
*e*_4_ stands for the parameter “cheap,”
 
*e*_5_ stands for the parameter “in the green surroundings.”

For a subset *A* = {*e*_1_, *e*_3_, *e*_4_}⊆*E*, consider the cubic soft set (*F*, *A*) with the tabular representation in Table 7.

For a subset *B* = {*e*_3_, *e*_5_}⊆*E*, consider the cubic soft set (*G*, *B*) with the tabular representation in Table 8.

Then the *R*-union (*F*, *A*) ⋓_*R*_ (*G*, *B*) of (*F*, *A*) and (*G*, *B*) is the soft set over *U* with the tabular representation in Table 9.

Note that (*F*, *A*) and (*G*, *B*) are internal cubic soft sets over *U*. But the *R*-union (*F*, *A*) ⋓_*R*_ (*G*, *B*) = (*H*, *A* ∪ *B*) is not an internal cubic soft set over *U* since $\lambda_{H(e_{3})}(h_{3})=0.4\notin [0.5,0.7]=\left[{\bar{\mu }}_{H(e_{3})}^{-}(h_{3}),{\bar{\mu }}_{H(e_{3})}^{+}(h_{3})\right]$.

Next, we provide a condition for the *R*-union of two internal cubic soft sets to be an internal cubic soft set.

**Theorem 11**

Let (*F*, *A*) and (*G*, *B*) be internal cubic soft sets over *U* such that

(26) $$\begin{array}{c} \max\left\{{\bar{\mu }}_{F(e)}^{-}\left(x\right),{\bar{\mu }}_{G(e)}^{-}\left(x\right)\right\}\leq \min\left\{\lambda_{F(e)}\left(x\right),\lambda_{G(e)}\left(x\right)\right\}, \end{array}$$

for all *e* ∈ *A*∩*B* and *x* ∈ *U*. Then the *R*-union (*F*, *A*) ⋓_*R*_ (*G*, *B*) = (*H*, *A* ∪ *B*) of (*F*, *A*) and (*G*, *B*) is an internal cubic soft set over *U*.

**Proof**

Since (*F*, *A*) and (*G*, *B*) are internal cubic soft sets over *U*, if *e* ∈ *A* or *e* ∈ *B*, then clearly the *R*-union (*F*, *A*) ⋓_*R*_ (*G*, *B*) = (*H*, *A* ∪ *B*) of (*F*, *A*) and (*G*, *B*) is an internal cubic soft set over *U*. Note that

(27) $$\begin{array}{c} {\bar{\mu }}_{F\left(e_{A}\right)}^{-}\left(x\right)\leq \lambda_{F(e_{A})}\left(x\right)\leq {\bar{\mu }}_{F\left(e_{A}\right)}^{+}\left(x\right),\\ {\bar{\mu }}_{G\left(e_{B}\right)}^{-}\left(x\right)\leq \lambda_{G(e_{B})}\left(x\right)\leq {\bar{\mu }}_{G\left(e_{B}\right)}^{+}\left(x\right), \end{array}$$

for all *e*_*A*_ ∈ *A*, *e*_*B*_ ∈ *B* and *x* ∈ *U*. It follows from (26) that

(28) max⁡{μ¯F(e)−(x),μ¯G(e)−(x)}≤min⁡{λF(e)(x),λG(e)(x)}≤max⁡{μ¯F(e)+(x),μ¯G(e)+(x)},

for all *e* ∈ *A*∩*B* and *x* ∈ *U*. Therefore the *R*-union (*F*, *A*) ⋓_*R*_ (*G*, *B*) = (*H*, *A* ∪ *B*) of (*F*, *A*) and (*G*, *B*) is an internal cubic soft set over *U*.

## 4. Cubic Soft Subalgebras of BCK/BCI-Algebras

In what follows, let *U* be an initial universe set which is a BCK/BCI-algebra.

**Definition 12 (see [29])**

A cubic soft set (*F*, *A*) over *U* is said to be a* cubic soft* BCK/BCI-algebra over *U* based on a parameter *ɛ* (briefly, *ɛ-cubic soft subalgebra* over *U*) if there exists a parameter *ɛ* ∈ *A* such that

(29) μ¯F(ɛ)(x∗y)⪰rmin⁡{μ¯F(ɛ)(x),μ¯F(ɛ)(y)},

(30) λF(ɛ)(x∗y)≤max⁡{λF(ɛ)(x),λF(ɛ)(y)}

for all *x*, *y* ∈ *U*. If (*F*, *A*) is an *ɛ*-cubic soft subalgebra over *U* for all *ɛ* ∈ *A*, one says that (*F*, *A*) is a* cubic soft subalgebra* over *U*.

**Definition 13 (see [29])**

The *R-union* of cubic soft sets (*F*, *A*) and (*G*, *B*) over *U* is a cubic soft set (*H*, *C*), where *C* = *A* ∪ *B* and

(31) H(e)={F(e)if  e∈A∖B,G(e)if  e∈B∖A,F(e)⋃RG(e)if  e∈A∩B,

for all *e* ∈ *C*. This is denoted by (*H*, *C*) = (*F*, *A*) ⋓_*R*_ (*G*, *B*).

**Theorem 14**

Let (*F*, *A*) and (*G*, *B*) be cubic soft subalgebras over *U*. If *A* and *B* are disjoint, then the *R*-union of (*F*, *A*) and (*G*, *B*) is a cubic soft subalgebra over *U*.

**Proof**

By means of Definition 13, we can write (*F*, *A*) ⋓_*R*_ (*G*, *B*) = (*H*, *C*), where *C* = *A* ∪ *B* and for all *e* ∈ *C*,

(32) H(e)={F(e)if  e∈A∖B,G(e)if  e∈B∖A,F(e)⋃RG(e)if  e∈A∩B.

Since *A*∩*B* = *∅*, either *e* ∈ *A*∖*B* or *e* ∈ *B*∖*A* for all *e* ∈ *C*. If *e* ∈ *A*∖*B*, then *H*(*e*) = *F*(*e*) is a cubic soft subalgebra over *U*. If *e* ∈ *B*∖*A*, then *H*(*e*) = *G*(*e*) is a cubic soft subalgebra over *U*. Hence (*H*, *C*) = (*F*, *A*) ⋓_*R*_ (*G*, *B*) is a cubic soft subalgebra over *U*.

The following example shows that Theorem 14 is not valid if *A* and *B* are not disjoint.

**Example 15**

Let

(33) $$\begin{array}{c} U∶=\left\{\text{white},\text{blackish},\text{reddish},\text{green},\text{yellow}\right\} \end{array}$$

be a universe, and consider a binary operation *✠* which produces the following products:

(34) $$\begin{array}{c} \text{white}\;\;✠\;x=\{\begin{array}{cc} \text{white} & \text{if}\;\;x\in \left\{\text{white, blackish}\right\},\\ \text{reddish} & \text{if}\;x=\text{reddish},\\ \text{green} & \text{if}\;\;x=\text{green},\\ \text{yellow} & \text{if}\;\;x=\text{yellow}, \end{array}\\ \text{blackish}\;\;✠\;y=\{\begin{array}{cc} \text{blackish} & \text{if}\;y=\text{white,}\\ \text{white} & \text{if}\;y=\text{blackish,}\\ \text{reddish} & \text{if}\;y=\text{reddish,}\\ \text{green} & \text{if}\;y=\text{green,}\\ \text{yellow} & \text{if}\;y=\text{yellow,} \end{array}\\ \text{reddish}\;\;✠\;z=\{\begin{array}{cc} \text{white} & \text{if}\;z=\text{reddish,}\\ \text{reddish} & \text{if}\;z\in \left\{\text{white, blackish}\right\},\\ \text{yellow} & \text{if}\;z=\text{green,}\\ \text{green} & \text{if}\;z=\text{yellow,} \end{array}\\ \text{green}\;\;✠\;u=\{\begin{array}{cc} \text{white} & \text{if}\;u=\text{green,}\\ \text{green} & \text{if}\;u\in \left\{\text{white, blackish}\right\}\text{,}\\ \text{yellow} & \text{if}\;u=\text{reddish,}\\ \text{reddish} & \text{if}\;u=\text{yellow,} \end{array}\\ \text{yellow}\;\;✠\;v=\{\begin{array}{cc} \text{white} & \text{if}\;v=\text{yellow,}\\ \text{reddish} & \text{if}\;v=\text{green,}\\ \text{green} & \text{if}\;v=\text{reddish,}\\ \text{yellow} & \text{if}\;v\in \left\{\text{white, blackish}\right\}. \end{array} \end{array}$$

Then, (*U*, *✠*, white) is a BCI-algebra (see [30]). Consider sets of parameters:

(35) $$\begin{array}{c} A∶=\left\{e_{1},e_{2},e_{3},e_{4}\right\},\\ B∶=\left\{e_{3},e_{4},e_{5}\right\}, \end{array}$$

where

 
*e*_1_ stands for the parameter “beautiful,”
 
*e*_2_ stands for the parameter “fine,”
 
*e*_3_ stands for the parameter “moderate,”
 
*e*_4_ stands for the parameter “smart,”
 
*e*_5_ stands for the parameter “chaste.”

Then *A* and *B* are not disjoint, and a soft set (*F*, *A*) over *U* with the tabular representation in Table 10 is a cubic soft subalgebra over *U*.

Also a soft set (*G*, *B*) over *U* with the tabular representation in Table 11 is a cubic soft subalgebra over *U*.

Then the *R*-union (*H*, *C*) = (*F*, *A*) ⋓_*R*_ (*G*, *B*) of (*F*, *A*) and (*G*, *B*) is represented by Table 12.

Note that

(36) μ¯H(e3)(green  ✠  reddish)  =μ¯H(e3)(yellow)=[0.1,0.4]  ⋡[0.3,0.4]=rmin⁡{[0.3,0.4],[0.3,0.4]}  =rmin⁡{μ¯H(e3)(green),μ¯H(e3)(reddish)}

and/or

(37) λH(e3)(green  ✠  reddish)  =λH(e3)(yellow)=0.9  ≰0.7=max⁡{λH(e3)(green),λH(e3)(reddish)}.

Therefore, the *R*-union (*H*, *C*) = (*F*, *A*) ⋓_*R*_ (*G*, *B*) of (*F*, *A*) and (*G*, *B*) is not a cubic soft subalgebra over *U*.

**Theorem 16**

Given a parameter *ɛ* ∈ *A*, a cubic soft set (*F*, *A*) over *U* is an *ɛ*-cubic soft subalgebra over *U* if and only if the sets

(38) $$\begin{array}{c} {\bar{\mu }}_{F(ɛ)}^{⇐}\left[\delta_{1},\delta_{2}\right]∶=\left\{x\in U\;|\;{\bar{\mu }}_{F(ɛ)}\left(x\right)⪰\left[\delta_{1},\delta_{2}\right]\right\},\\ \lambda_{F\left(ɛ\right)}^{\to }\left(t\right)∶=\left\{x\in U\;|\;\lambda_{F(ɛ)}\left(x\right)\leq t\right\} \end{array}$$

are subalgebras of *U* for all [*δ*_1_, *δ*_2_]∈[*I*] and *t* ∈ [0,1].

**Proof**

Assume that a cubic soft set (*F*, *A*) over *U* is an *ɛ*-cubic soft subalgebra over *U* and let *x*, *y* ∈ *U*. If $x,y\in {\bar{\mu }}_{F(ɛ)}^{⇐}[\delta_{1},\delta_{2}]$ for every [*δ*_1_, *δ*_2_]∈[*I*], then ${\bar{\mu }}_{F(ɛ)}(x)⪰[\delta_{1},\delta_{2}]$ and ${\bar{\mu }}_{F(ɛ)}(y)⪰[\delta_{1},\delta_{2}]$. It follows from (29) that

(39) μ¯F(ɛ)(x∗y)⪰rmin⁡{μ¯F(ɛ)(x),μ¯F(ɛ)(y)}⪰rmin⁡{[δ1,δ2],[δ1,δ2]}=[δ1,δ2].

Hence $x*y\in {\bar{\mu }}_{F(ɛ)}^{⇐}[\delta_{1},\delta_{2}]$. Now if *x*, *y* ∈ *λ*_*F*(*ɛ*)_^→^(*t*) for all *t* ∈ [0,1], then *λ*_*F*(*ɛ*)_(*x*) ≤ *t* and *λ*_*F*(*ɛ*)_(*y*) ≤ *t*. Using (30), we have *λ*_*F*(*ɛ*)_(*x*∗*y*) ≤ max⁡{*λ*_*F*(*ɛ*)_(*x*), *λ*_*F*(*ɛ*)_(*y*)} ≤ *t*, and so *x*∗*y* ∈ *λ*_*F*(*ɛ*)_^→^(*t*). Therefore ${\bar{\mu }}_{F(ɛ)}^{⇐}[\delta_{1},\delta_{2}]$ and *λ*_*F*(*ɛ*)_^→^(*t*) are subalgebras of *U*.

Conversely, suppose that ${\bar{\mu }}_{F(ɛ)}^{⇐}[\delta_{1},\delta_{2}]$ and *λ*_*F*(*ɛ*)_^→^(*t*) are subalgebras of *U* for all [*δ*_1_, *δ*_2_]∈[*I*] and *t* ∈ [0,1]. Assume that there exists *a*, *b* ∈ *U* such that

(40) $$\begin{array}{c} {\bar{\mu }}_{F(ɛ)}\left(a*b\right)⋡r\min\left\{{\bar{\mu }}_{F(ɛ)}\left(a\right),{\bar{\mu }}_{F(ɛ)}\left(b\right)\right\}. \end{array}$$

Let ${\bar{\mu }}_{F(ɛ)}(a)=[\gamma_{1},\gamma_{2}]$, ${\bar{\mu }}_{F(ɛ)}(b)=[\gamma_{3},\gamma_{4}]$, and ${\bar{\mu }}_{F(ɛ)}(a*b)=[\delta_{1},\delta_{2}]$. Then

(41) [δ1,δ2]≺rmin⁡{[γ1,γ2],[γ3,γ4]}=[min⁡{γ1,γ3},min⁡{γ2,γ4}].

Hence, *δ*_1_ < min⁡{*γ*_1_, *γ*_3_} and *δ*_2_ < min⁡{*γ*_2_, *γ*_4_}. Taking

(42) $$\begin{array}{c} \left[\tau_{1},\tau_{2}\right]=\frac{1}{2}\left({\bar{\mu }}_{F(ɛ)}\left(a*b\right)+r\min\left\{{\bar{\mu }}_{F(ɛ)}\left(a\right),{\bar{\mu }}_{F(ɛ)}\left(b\right)\right\}\right) \end{array}$$

implies that

(43) [τ1,τ2]=12([δ1,δ2]+[min⁡{γ1,γ3},min⁡{γ2,γ4}])=[12(δ1+min⁡{γ1,γ3}),12(δ2+min⁡{γ2,γ4})].

It follows that

(44) min⁡{γ1,γ3}>τ1=12(δ1+min⁡{γ1,γ3})>δ1,min⁡{γ2,γ4}>τ2=12(δ2+min⁡{γ2,γ4})>δ2,

and so that

(45) [min⁡{γ1,γ3},min⁡{γ2,γ4}]≻[τ1,τ2]≻[δ1,δ2]=μ¯F(ɛ)(a∗b).

Therefore $a*b\notin {\bar{\mu }}_{F(ɛ)}^{⇐}[\tau_{1},\tau_{2}]$. On the other hand, we know that

(46) μ¯F(ɛ)(a)=[γ1,γ2]⪰[min⁡⁡{γ1,γ3},min⁡⁡{γ2,γ4}]≻[τ1,τ2],μ¯F(ɛ)(b)=[γ3,γ4]⪰[min⁡⁡{γ1,γ3},min⁡⁡{γ2,γ4}]≻[τ1,τ2],

which imply that $a,b\in {\bar{\mu }}_{F(ɛ)}^{⇐}[\tau_{1},\tau_{2}]$. This is a contradiction, and so

(47) $$\begin{array}{c} {\bar{\mu }}_{F(ɛ)}\left(x*y\right)⪰r\min\left\{{\bar{\mu }}_{F(ɛ)}\left(x\right),{\bar{\mu }}_{F(ɛ)}\left(y\right)\right\} \end{array}$$

for all *x*, *y* ∈ *U*. Now, assume that *λ*_*F*(*ɛ*)_(*a*∗*b*) > max⁡{*λ*_*F*(*ɛ*)_(*a*), *λ*_*F*(*ɛ*)_(*b*)} for some *a*, *b* ∈ *U*. Then there exists *t*_0_ ∈ (0,1) such that

(48) $$\begin{array}{c} \lambda_{F(ɛ)}\left(a*b\right)\geq t_{0}>\max\left\{\lambda_{F(ɛ)}\left(a\right),\lambda_{F(ɛ)}\left(b\right)\right\}. \end{array}$$

Hence, *a*, *b* ∈ *λ*_*F*(*ɛ*)_^→^(*t*_0_) but *a*∗*b* ∉ *λ*_*F*(*ɛ*)_^→^(*t*_0_). This is a contradiction, and therefore

(49) $$\begin{array}{c} \lambda_{F(ɛ)}\left(x*y\right)\leq \max\left\{\lambda_{F(ɛ)}\left(x\right),\lambda_{F(ɛ)}\left(y\right)\right\} \end{array}$$

for all *x*, *y* ∈ *U*. Consequently, (*F*, *A*) is an *ɛ*-cubic soft subalgebra over *U*.

**Proposition 17**

Given a parameter *ɛ* ∈ *A*, if a cubic soft set (*F*, *A*) over *U* is an *ɛ*-cubic soft subalgebra over *U*, then ${\bar{\mu }}_{F(ɛ)}(0)⪰{\bar{\mu }}_{F(ɛ)}(x)$ and *λ*_*F*(*ɛ*)_(0) ≤ *λ*_*F*(*ɛ*)_(*x*), for all *x* ∈ *U*.

**Proof**

For every *x* ∈ *U*, we have

(50) μ¯F(ɛ)(0)  =μ¯F(ɛ)(x∗x)⪰rmin⁡{μ¯F(ɛ)(x),μ¯F(ɛ)(x)}  =r min⁡{[μ¯F(ɛ)−(x),μ¯F(ɛ)+(x)],[μ¯F(ɛ)−(x),μ¯F(ɛ)+(x)]}  =[μ¯F(ɛ)−(x),μ¯F(ɛ)+(x)]=μ¯F(ɛ)(x)

and *λ*_*F*(*ɛ*)_(0) = *λ*_*F*(*ɛ*)_(*x*∗*x*) ≤ max⁡{*λ*_*F*(*ɛ*)_(*x*), *λ*_*F*(*ɛ*)_(*x*)} = *λ*_*F*(*ɛ*)_(*x*).

**Theorem 18**

Let (*F*, *A*) be an *ɛ*-cubic soft subalgebra over *U* for a parameter *ɛ* ∈ *A*. If there is a sequence in *U* such that $\lim_{n\to \infty }{\bar{\mu }}_{F(ɛ)}(x_{n})=[1,1]$ and lim⁡_*n*→*∞*_ *λ*_*F*(*ε*)_(*x*_*n*_) = 0, then ${\bar{\mu }}_{F(ɛ)}(0)=[1,1]$ and *λ*_*F*(*ɛ*)_(0) = 0.

**Proof**

Since ${\bar{\mu }}_{F(ɛ)}(0)⪰{\bar{\mu }}_{F(ɛ)}(x)$ and *λ*_*F*(*ɛ*)_(0) ≤ *λ*_*F*(*ɛ*)_(*x*), for all *x* ∈ *U*, we have

(51) $$\begin{array}{c} {\bar{\mu }}_{F(ɛ)}\left(0\right)⪰{\bar{\mu }}_{F(ɛ)}\left(x_{n}\right),\\ \lambda_{F(ɛ)}\left(0\right)\leq \lambda_{F(ɛ)}\left(x_{n}\right), \end{array}$$

for every positive integer *n*. Note that $[1,1]⪰{\bar{\mu }}_{F(ɛ)}(0)⪰\lim_{n\to \infty }{\bar{\mu }}_{F(ɛ)}(x_{n})=[1,1]$ and 0 ≤ *λ*_*F*(*ɛ*)_(0) ≤ *λ*_*F*(*ɛ*)_(*x*_*n*_) = 0. Hence ${\bar{\mu }}_{F(ɛ)}(0)=[1,1]$ and *λ*_*F*(*ɛ*)_(0) = 0.

**Theorem 19**

Given a parameter *ɛ* ∈ *A*, if a cubic soft set (*F*, *A*) over *U* is an *ɛ*-cubic soft subalgebra over *U*, then the sets $U_{{\bar{\mu }}_{F(ɛ)}}∶=\{x\in U|{\bar{\mu }}_{F(ɛ)}(x)={\bar{\mu }}_{F(ɛ)}(0)\}$ and *U*_*λ*_*F*(*ɛ*)__∶ = {*x* ∈ *U* | *λ*_*F*(*ɛ*)_(*x*) = *λ*_*F*(*ɛ*)_(0)} are subalgebras of *U*.

**Proof**

Let *x*, *y* ∈ *U*. If $x,y\in U_{{\bar{\mu }}_{F(ɛ)}}$, then ${\bar{\mu }}_{F(ɛ)}(x)={\bar{\mu }}_{F(ɛ)}(0)={\bar{\mu }}_{F(ɛ)}(y)$. Hence,

(52) μ¯F(ɛ)(x∗y)⪰rmin⁡{μ¯F(ɛ)(x),μ¯F(ɛ)(y)}=rmin⁡{μ¯F(ɛ)(0),μ¯F(ɛ)(0)}=μ¯F(ɛ)(0)

and *λ*_*F*(*ɛ*)_(*x*∗*y*) ≤ max⁡{*λ*_*F*(*ɛ*)_(*x*), *λ*_*F*(*ɛ*)_(*y*)} = max⁡{*λ*_*F*(*ɛ*)_(0), *λ*_*F*(*ɛ*)_(0)} = *λ*_*F*(*ɛ*)_(0). Combining this and Proposition 17, we have ${\bar{\mu }}_{F(ɛ)}(x*y)={\bar{\mu }}_{F(ɛ)}(0)$ and *λ*_*F*(*ɛ*)_(*x*∗*y*) = *λ*_*F*(*ɛ*)_(0). This shows that $x*y\in U_{{\bar{\mu }}_{F(ɛ)}}$ and *x*∗*y* ∈ *U*_*λ*_*F*(*ɛ*)__. Therefore $U_{{\bar{\mu }}_{F(ɛ)}}$ and *U*_*λ*_*F*(*ɛ*)__ are subalgebras of *U*.

**Corollary 20**

Given a parameter *ɛ* ∈ *A*, if a cubic soft set (*F*, *A*) over *U* is an *ɛ*-cubic soft subalgebra over *U*, then the set $U_{{\bar{\mu }}_{F(ɛ)}}\cap U_{\lambda_{F(ɛ)}}$ is a subalgebra of *U*.

**Proof**

The proof is straightforward.

## 5. Conclusion

In this paper, we first have considered operations of cubic soft sets, that is, “AND” operation and “OR” operation based on the *P*-order and the *R*-order. In [29], Muhiuddin and Al-roqi have posed a question: is the *R*-union of two internal cubic soft sets an internal cubic soft set? We have given an example to show that the answer to this question is negative, and then we have provided a condition for the *R*-union of two internal cubic soft sets to be an internal cubic soft set. We also have investigated several properties of cubic soft subalgebras of BCK/BCI-algebras based on any given parameter. Some important issues to be explored in the future include

1. developing strategies for obtaining more valuable results,
2. applying these notions and results for studying related notions in other (soft) algebraic structures.

## Figures and Tables

**Table 1:** Tabular representation of the cubic soft set (*F*, *A*).

	*e*_1_	*e*_3_	*e*_4_
*h*_1_	〈[0.5,0.8], 0.6〉	〈[0.2,0.5], 0.3〉	〈[0.4,0.6], 0.7〉
*h*_2_	〈[1.0,1.0], 0.7〉	〈[0.3,0.6], 0.7〉	〈[0.1,0.2], 0.7〉
*h*_3_	〈[0.1,0.7], 0.5〉	〈[0.1,0.2], 0.4〉	〈[0.1,0.7], 0.3〉
*h*_4_	〈[0.2,0.6], 0.9〉	〈[0.2,0.7], 0.2〉	〈[0.3,0.6], 0.2〉
*h*_5_	〈[0.3,0.9], 0.4〉	〈[0.7,0.9], 0.5〉	〈[0.4,0.8], 0.7〉
*h*_6_	〈[0.2,0.3], 0.3〉	〈[0.3,0.5], 0.3〉	〈[0.6,0.7], 0.8〉

**Table 2:** Tabular representation of the cubic soft set (*G*, *B*).

	*e*_2_	*e*_5_
*h*_1_	〈[0.3,0.7], 0.5〉	〈[0.4,0.6], 0.4〉
*h*_2_	〈[0.2,0.6], 0.7〉	〈[0.5,0.8], 0.6〉
*h*_3_	〈[0.5,0.7], 0.3〉	〈[0.1,0.7], 0.3〉
*h*_4_	〈[0.8,0.9], 0.6〉	〈[0.3,0.5], 0.8〉
*h*_5_	〈[0.4,0.5], 0.2〉	〈[0.7,0.9], 0.5〉
*h*_6_	〈[0.1,0.3], 0.9〉	〈[0.2,0.8], 0.9〉

**Table 3:** Tabular representation of the cubic soft set $\left(F,A){\tilde{\vee }}_{P}(G,B)=(H,A\times B\right)$.

	(*e*_1_, *e*_2_)	(*e*_1_, *e*_5_)	(*e*_3_, *e*_2_)	(*e*_3_, *e*_5_)	(*e*_4_, *e*_2_)	(*e*_4_, *e*_5_)
*h*_1_	〈[0.5,0.8], 0.6〉	〈[0.5,0.8], 0.6〉	〈[0.3,0.7], 0.5〉	〈[0.4,0.6], 0.4〉	〈[0.4,0.7], 0.7〉	〈[0.4,0.6], 0.7〉
*h*_2_	〈[1.0,1.0], 0.7〉	〈[1.0,1.0], 0.7〉	〈[0.3,0.6], 0.7〉	〈[0.5,0.8], 0.7〉	〈[0.2,0.6], 0.7〉	〈[0.5,0.8], 0.7〉
*h*_3_	〈[0.5,0.7], 0.5〉	〈[0.1,0.7], 0.5〉	〈[0.5,0.7], 0.4〉	〈[0.1,0.7], 0.4〉	〈[0.5,0.7], 0.3〉	〈[0.1,0.7], 0.3〉
*h*_4_	〈[0.8,0.9], 0.9〉	〈[0.3,0.6], 0.9〉	〈[0.8,0.9], 0.6〉	〈[0.3,0.7], 0.8〉	〈[0.8,0.9], 0.6〉	〈[0.3,0.6], 0.8〉
*h*_5_	〈[0.4,0.9], 0.4〉	〈[0.7,0.9], 0.5〉	〈[0.7,0.9], 0.5〉	〈[0.7,0.9], 0.5〉	〈[0.4,0.8], 0.7〉	〈[0.7,0.9], 0.7〉
*h*_6_	〈[0.2,0.3], 0.9〉	〈[0.2,0.8], 0.9〉	〈[0.3,0.5], 0.9〉	〈[0.3,0.8], 0.9〉	〈[0.6,0.7], 0.9〉	〈[0.6,0.8], 0.9〉

**Table 4:** Tabular representation of the cubic soft set $\left(F,A){\tilde{\vee }}_{R}(G,B)=(H,A\times B\right)$.

	(*e*_1_, *e*_2_)	(*e*_1_, *e*_5_)	(*e*_3_, *e*_2_)	(*e*_3_, *e*_5_)	(*e*_4_, *e*_2_)	(*e*_4_, *e*_5_)
*h*_1_	〈[0.5,0.8], 0.5〉	〈[0.5,0.8], 0.4〉	〈[0.3,0.7], 0.3〉	〈[0.4,0.6], 0.3〉	〈[0.4,0.7], 0.5〉	〈[0.4,0.6], 0.4〉
*h*_2_	〈[1.0,1.0], 0.7〉	〈[1.0,1.0], 0.6〉	〈[0.3,0.6], 0.7〉	〈[0.5,0.8], 0.6〉	〈[0.2,0.6], 0.7〉	〈[0.5,0.8], 0.6〉
*h*_3_	〈[0.5,0.7], 0.3〉	〈[0.1,0.7], 0.3〉	〈[0.5,0.7], 0.3〉	〈[0.1,0.7], 0.3〉	〈[0.5,0.7], 0.3〉	〈[0.1,0.7], 0.3〉
*h*_4_	〈[0.8,0.9], 0.6〉	〈[0.3,0.6], 0.8〉	〈[0.8,0.9], 0.2〉	〈[0.3,0.7], 0.2〉	〈[0.8,0.9], 0.2〉	〈[0.3,0.6], 0.2〉
*h*_5_	〈[0.4,0.9], 0.2〉	〈[0.7,0.9], 0.4〉	〈[0.7,0.9], 0.2〉	〈[0.7,0.9], 0.5〉	〈[0.4,0.8], 0.2〉	〈[0.7,0.9], 0.5〉
*h*_6_	〈[0.2,0.3], 0.3〉	〈[0.2,0.8], 0.3〉	〈[0.3,0.5], 0.3〉	〈[0.3,0.8], 0.3〉	〈[0.6,0.7], 0.8〉	〈[0.6,0.8], 0.8〉

**Table 5:** Tabular representation of the cubic soft set $\left(F,A){\tilde{\wedge }}_{P}(G,B)=(H,A\times B\right)$.

	(*e*_1_, *e*_2_)	(*e*_1_, *e*_5_)	(*e*_3_, *e*_2_)	(*e*_3_, *e*_5_)	(*e*_4_, *e*_2_)	(*e*_4_, *e*_5_)
*h*_1_	〈[0.3,0.7], 0.5〉	〈[0.4,0.6], 0.4〉	〈[0.2,0.5], 0.3〉	〈[0.2,0.5], 0.3〉	〈[0.3,0.6], 0.5〉	〈[0.4,0.6], 0.4〉
*h*_2_	〈[0.2,0.6], 0.7〉	〈[0.5,0.8], 0.6〉	〈[0.2,0.6], 0.7〉	〈[0.3,0.6], 0.6〉	〈[0.1,0.2], 0.7〉	〈[0.1,0.2], 0.6〉
*h*_3_	〈[0.1,0.7], 0.3〉	〈[0.1,0.7], 0.3〉	〈[0.1,0.2], 0.3〉	〈[0.1,0.2], 0.3〉	〈[0.1,0.7], 0.3〉	〈[0.1,0.7], 0.3〉
*h*_4_	〈[0.2,0.6], 0.6〉	〈[0.2,0.5], 0.8〉	〈[0.2,0.7], 0.2〉	〈[0.2,0.5], 0.2〉	〈[0.3,0.6], 0.2〉	〈[0.3,0.5], 0.2〉
*h*_5_	〈[0.3,0.5], 0.2〉	〈[0.3,0.9], 0.4〉	〈[0.4,0.5], 0.2〉	〈[0.7,0.9], 0.5〉	〈[0.4,0.5], 0.2〉	〈[0.4,0.8], 0.5〉
*h*_6_	〈[0.1,0.3], 0.3〉	〈[0.2,0.3], 0.3〉	〈[0.1,0.3], 0.3〉	〈[0.2,0.5], 0.3〉	〈[0.1,0.3], 0.8〉	〈[0.2,0.7], 0.8〉

**Table 6:** Tabular representation of the cubic soft set $\left(F,A){\tilde{\wedge }}_{R}(G,B)=(H,A\times B\right)$.

	(*e*_1_, *e*_2_)	(*e*_1_, *e*_5_)	(*e*_3_, *e*_2_)	(*e*_3_, *e*_5_)	(*e*_4_, *e*_2_)	(*e*_4_, *e*_5_)
*h*_1_	〈[0.3,0.7], 0.6〉	〈[0.4,0.6], 0.6〉	〈[0.2,0.5], 0.5〉	〈[0.2,0.5], 0.4〉	〈[0.3,0.6], 0.7〉	〈[0.4,0.6], 0.7〉
*h*_2_	〈[0.2,0.6], 0.7〉	〈[0.5,0.8], 0.7〉	〈[0.2,0.6], 0.7〉	〈[0.3,0.6], 0.7〉	〈[0.1,0.2], 0.7〉	〈[0.1,0.2], 0.7〉
*h*_3_	〈[0.1,0.7], 0.5〉	〈[0.1,0.7], 0.5〉	〈[0.1,0.2], 0.4〉	〈[0.1,0.2], 0.4〉	〈[0.1,0.7], 0.3〉	〈[0.1,0.7], 0.3〉
*h*_4_	〈[0.2,0.6], 0.9〉	〈[0.2,0.5], 0.9〉	〈[0.2,0.7], 0.6〉	〈[0.2,0.5], 0.8〉	〈[0.3,0.6], 0.6〉	〈[0.3,0.5], 0.8〉
*h*_5_	〈[0.3,0.5], 0.4〉	〈[0.3,0.9], 0.5〉	〈[0.4,0.5], 0.5〉	〈[0.7,0.9], 0.5〉	〈[0.4,0.5], 0.7〉	〈[0.4,0.8], 0.7〉
*h*_6_	〈[0.1,0.3], 0.9〉	〈[0.2,0.3], 0.9〉	〈[0.1,0.3], 0.9〉	〈[0.2,0.5], 0.9〉	〈[0.1,0.3], 0.9〉	〈[0.2,0.7], 0.9〉

**Table 7:** Tabular representation of the cubic soft set (*F*, *A*).

	*e*_1_	*e*_3_	*e*_4_
*h*_1_	〈[0.5,0.8], 0.6〉	〈[0.2,0.5], 0.3〉	〈[0.4,0.6], 0.6〉
*h*_2_	〈[0.3,0.5], 0.4〉	〈[0.3,0.6], 0.5〉	〈[0.1,0.2], 0.2〉
*h*_3_	〈[0.1,0.7], 0.5〉	〈[0.1,0.5], 0.4〉	〈[0.1,0.7], 0.3〉

**Table 8:** Tabular representation of the cubic soft set (*G*, *B*).

	*e*_3_	*e*_5_
*h*_1_	〈[0.3,0.7], 0.5〉	〈[0.4,0.6], 0.4〉
*h*_2_	〈[0.2,0.6], 0.6〉	〈[0.5,0.8], 0.6〉
*h*_3_	〈[0.5,0.7], 0.6〉	〈[0.1,0.7], 0.3〉

**Table 9:** Tabular representation of the cubic soft set (*F*, *A*)⋓_*R*_(*G*, *B*) = (*H*, *A* ∪ *B*).

	*e*_1_	*e*_3_	*e*_4_	*e*_5_
*h*_1_	〈[0.5,0.8], 0.6〉	〈[0.3,0.7], 0.3〉	〈[0.4,0.6], 0.6〉	〈[0.4,0.6], 0.4〉
*h*_2_	〈[0.3,0.5], 0.4〉	〈[0.3,0.6], 0.5〉	〈[0.1,0.2], 0.2〉	〈[0.5,0.8], 0.6〉
*h*_3_	〈[0.1,0.7], 0.5〉	〈[0.5,0.7], 0.4〉	〈[0.1,0.7], 0.3〉	〈[0.1,0.7], 0.3〉

**Table 10:** Tabular representation of the cubic soft set (*F*, *A*).

	*e*_1_	*e*_2_	*e*_3_	*e*_4_
White	〈[0.6,0.8], 0.3〉	〈[0.4,0.6], 0.4〉	〈[0.7,0.9], 0.2〉	〈[0.2,0.5], 0.5〉
Blackish	〈[0.7,0.8], 0.4〉	〈[0.6,0.7], 0.5〉	〈[0.6,0.7], 0.5〉	〈[0.6,0.7], 0.5〉
Reddish	〈[0.3,0.4], 0.7〉	〈[0.5,0.7], 0.6〉	〈[0.1,0.4], 0.9〉	〈[0.2,0.5], 0.8〉
Green	〈[0.3,0.4], 0.7〉	〈[0.2,0.5], 0.8〉	〈[0.3,0.4], 0.7〉	〈[0.2,0.5], 0.8〉
Yellow	〈[0.3,0.4], 0.7〉	〈[0.2,0.5], 0.8〉	〈[0.1,0.4], 0.9〉	〈[0.5,0.7], 0.6〉

**Table 11:** Tabular representation of the cubic soft set (*G*, *B*).

	*e*_3_	*e*_4_	*e*_5_
White	〈[0.8,0.9], 0.2〉	〈[0.7,0.8], 0.3〉	〈[0.9,1.0], 0.1〉
Blackish	〈[0.6,0.7], 0.4〉	〈[0.6,0.7], 0.4〉	〈[0.5,0.6], 0.5〉
Reddish	〈[0.3,0.4], 0.7〉	〈[0.3,0.4], 0.7〉	〈[0.2,0.3], 0.8〉
Green	〈[0.1,0.2], 0.9〉	〈[0.3,0.4], 0.7〉	〈[0.4,0.5], 0.6〉
Yellow	〈[0.1,0.2], 0.9〉	〈[0.5,0.6], 0.5〉	〈[0.2,0.3], 0.8〉

**Table 12:** Tabular representation of the cubic soft set (*H*, *C*).

	*e*_1_	*e*_2_	*e*_3_	*e*_4_	*e*_5_
White	〈[0.6,0.8], 0.3〉	〈[0.4,0.6], 0.4〉	〈[0.8,0.9], 0.2〉	〈[0.7,0.8], 0.3〉	〈[0.9,1.0], 0.1〉
Blackish	〈[0.7,0.8], 0.4〉	〈[0.6,0.7], 0.5〉	〈[0.6,0.7], 0.4〉	〈[0.6,0.7], 0.4〉	〈[0.5,0.6], 0.5〉
Reddish	〈[0.3,0.4], 0.7〉	〈[0.5,0.7], 0.6〉	〈[0.3,0.4], 0.7〉	〈[0.3,0.5], 0.7〉	〈[0.2,0.3], 0.8〉
Green	〈[0.3,0.4], 0.7〉	〈[0.2,0.5], 0.8〉	〈[0.3,0.4], 0.7〉	〈[0.3,0.5], 0.7〉	〈[0.4,0.5], 0.6〉
Yellow	〈[0.3,0.4], 0.7〉	〈[0.2,0.5], 0.8〉	〈[0.1,0.4], 0.9〉	〈[0.5,0.7], 0.5〉	〈[0.2,0.3], 0.8〉
